# A Nomogram for Predicting Risk of Thromboembolism in Gastric Cancer Patients Receiving Chemotherapy

**DOI:** 10.3389/fonc.2021.598116

**Published:** 2021-05-26

**Authors:** Hai-Liang Yuan, Xiang Zhang, Yan Li, Qing Guan, Wei-Wei Chu, Hai-Ping Yu, Lian Liu, Yun-Quan Zheng, Jing-Jing Lu

**Affiliations:** ^1^Department of Gastroenterology, Beilun Branch of the First Affiliated Hospital of Zhejiang University, Ningbo, China; ^2^Department of Gastroenterology Oncology, The First Affiliated Hospital of Nanchang University, Nanchang, China

**Keywords:** thromboembolism, gastric cancer, chemotherapy, nomogram, prediction

## Abstract

**Purpose:** The aims of this study were to develop and validate a novel nomogram to predict thromboembolism (TE) in gastric cancer (GC) patients receiving chemotherapy and to test its predictive ability.

**Methods:** This retrospective study included 544 GC patients who received chemotherapy as the initial treatment at two medical centers. Among the 544 GC patients who received chemotherapy, 275 and 137 patients in the First Affiliated Hospital of Nanchang University from January 2014 to March 2019 were enrolled in the training cohort and the validation cohort, respectively. A total of 132 patients in the Beilun branch of the First Affiliated Hospital of Zhejiang University from January 2015 to August 2019 were enrolled in external validation cohorts. The nomogram was based on parameters determined by univariate and multivariate logistic analyses. The prediction performance of the nomogram was measured by the area under the receiver operating characteristic curve (AUROC), the calibration curve, and decision curve analysis (DCA). The applicability of the nomogram was internally and independently validated.

**Results:** The predictors included the Eastern Cooperative Oncology Group Performance Status (ECOG), presence of an active cancer (AC), central venous catheter (CVC), and D-dimer levels. These risk factors are shown on the nomogram and verified. The nomogram demonstrated good discrimination and fine calibration with an AUROC of 0.875 (0.832 in internal validation and 0.807 in independent validation). The DCA revealed that the nomogram had a high clinical application value.

**Conclusions:** We propose the nomogram for predicting TE in patients with GC receiving chemotherapy, which can help in making timely personalized clinical decisions for different risk populations.

## Introduction

Thromboembolism (TE) is a common complication of malignant tumors, with an incidence of up to 20% in cancer patients (1), and is usually accidentally diagnosed during cancer treatment (2, 3). Cancer-associated TE is a common condition, which includes thromboembolism (VTE), arterial thromboembolism (ATE), and pulmonary embolism (PE). Cancer-associated TE, whether symptomatic or incidental, is a significant predictor of poor prognosis (4). For example, the occurrence of cancer-associated VTE is a significant predictor of death within 1 year of cancer diagnosis (5). In addition, TE is one of the leading causes of death in cancer patients receiving chemotherapy (6), and TE diagnosis can delay or interrupt chemotherapy initiation (6). TE occurring during antineoplastic treatment is a preventable complication causing a high economic burden (7). Therefore, early detection of high-risk factors for malignant tumors combined with TE is clinically significant and helps to improve the quality of life and prolong the survival in these patients.

Gastric cancer (GC) is one of the most common malignancies in the world and one of the common causes of cancer-related death (8, 9). Surgery is the main treatment for patients with early gastric cancer, while neoadjuvant/adjuvant chemotherapy and radiotherapy can improve the prognosis of advanced gastric cancer (8, 9). Among various cancer types, GC is a malignant diseases at high risk for TE (10). TE is a serious complication in GC patients undergoing chemotherapy. Preventing the occurrence of TE is very important since it is associated with huge medical and economic costs. Although prophylactic anticoagulant therapy can be used, there is an inherent risk of bleeding which may offset its clinical benefits. Therefore, there is an urgent need for new tools to accurately predict the risk of TE in patients with GC undergoing chemotherapy and to assess the benefits of prophylactic anticoagulant therapy.

In recent years, the nomogram is a simple and personalized visualization tool, which has been widely used in the diagnosis and prognosis of diseases (11). The nomogram is a complex calculation formula, which integrates multiple prediction indexes and then uses the line with scale to draw on the same plane according to a certain proportion, so that the prediction probability can be simply determined. Some studies have reported that nomogram prediction models have good value in disease diagnosis (12–14). In addition, the nomogram has been used to predict the risk of thromboembolism in cancer patients. For example, a recent study reported the application of nomogram in the risk of VTE in hospitalized patients with post-operative breast cancer (15). However, there is no report on using nomogram to predict venous thrombosis in gastric cancer patients receiving chemotherapy.

A predictive model is needed to determine the risk of TE in patients with GC undergoing chemotherapy in order to reduce the possibility of current overtreatment and not alter the prognosis of patients. The aim of this study was to establish a new predictive model for the probability of TE in patients with GC receiving chemotherapy, which can help determine the occurrence of TE and provide personalized early anticoagulant therapy strategies.

## Patients and Methods

### Patients

We retrospectively collected GC patients who received chemotherapy in the First Affiliated Hospital of Nanchang University from January 2014 to March 2019. Clinical data including age, gender, histological subtype, primary lesion resection, cancer type, and the Eastern Cooperative Oncology Group performance status (ECOG PS) scale were collected by viewing electronic medical records and using adjuvant chemotherapy and single or multiple main veins and by central vein catheter (CVC) placement. We selected cases clearly diagnosed as primary GC and receiving chemotherapy.

The inclusion criteria were 1) all primary gastric malignant tumors confirmed by pathological examination and 2) TE diagnosed by ultrasound or CT/MRI (16).

The exclusion criteria were as follows: 1) incomplete data; 2) TE occurring before chemotherapy; 3) those who had taken anticoagulant drugs within 1 month before chemotherapy; 4) prophylactic anticoagulation before TE occurring during chemotherapy; and 5) concomitant diseases such as atrial fibrillation, abnormal liver and kidney function, and malignant blood diseases.

**Figure 1:**
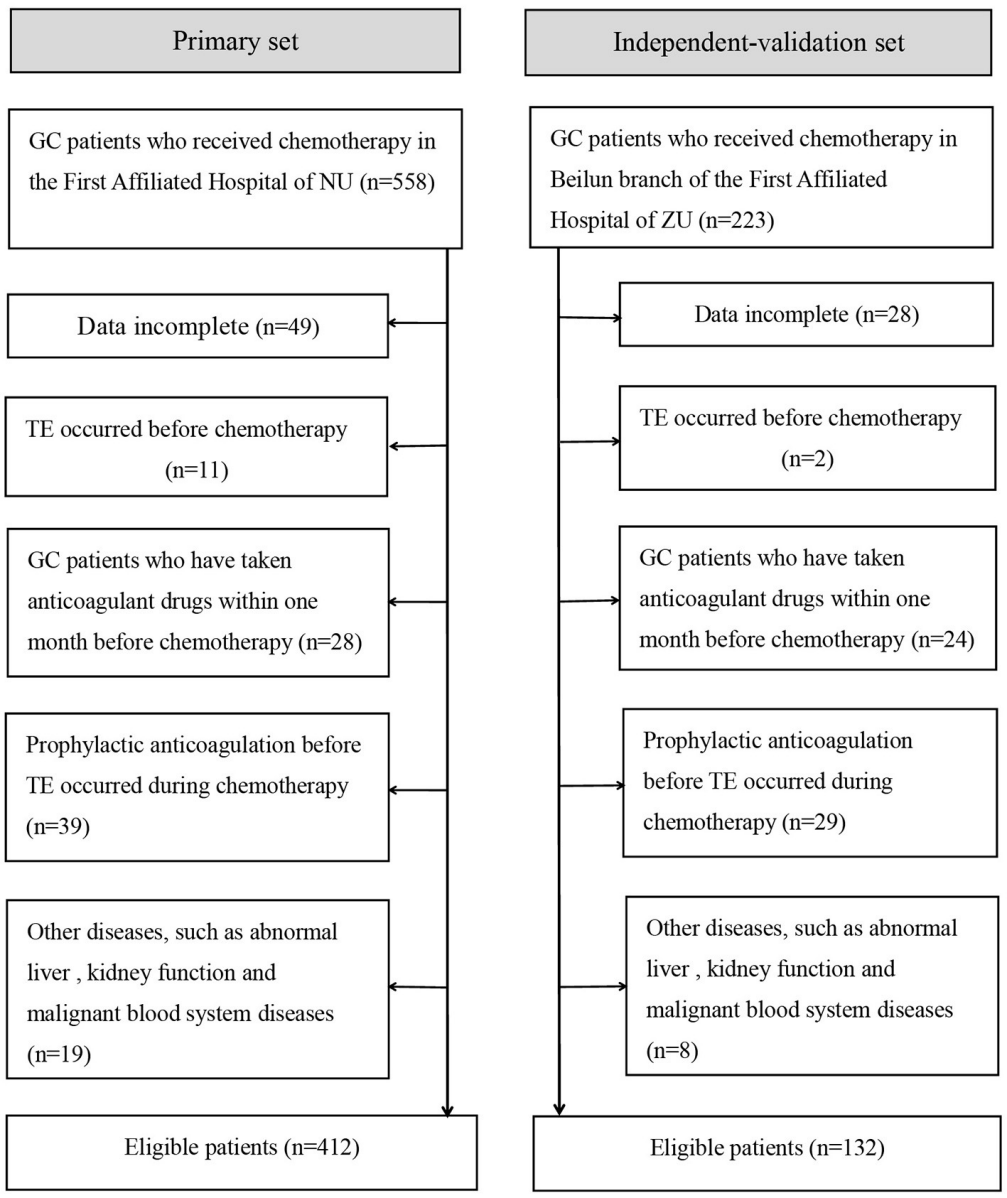
Flowchart of inclusion and exclusion of GC patients who received chemotherapy. NU, Nanchang University; ZU, Zhejiang University.

From January 2015 to August 2019, an independent validation study was conducted on GC patients who received chemotherapy in the Beilun branch of the First Affiliated Hospital of Zhejiang University using the same standards as the primary study. Figure 1 summarizes the patient inclusion/exclusion process.

### Statistical Analysis

SPSS version 25.0 (IBM, USA) and R software (version 3.6.1; https://www.r-project.org/) were used for statistical analysis. Continuous variables were expressed as mean and standard deviation (mean ± SD). Independent sample *t-*test or one-way ANOVA was used to compare differences. A *P* < 0.05 was considered statistically significant, and all tests were two-tailed unless otherwise indicated. For continuous variables, data were presented as median and interquartile range [*M* (P25, P75)] or mean ± SD. Categorical variables were presented as whole numbers and proportions.

### Construction and Validation of the Nomogram

Patients with GC receiving chemotherapy in the primary study were randomly divided into a training and internal validation group with a proportion of 2:1. Through binary multiple logistic regression analysis, a model was developed in the training dataset (17). Internal validation and independent validation were performed in the internal validation dataset and independent validation dataset, respectively. The logistic regression formula from the training set was used in all the patients in the internal and external validation sets, and the probability risk of TE in each GC patient was calculated.

Univariate and multivariate logistic regression analyses were used to calculate and validate the effect of variables in the training, internal validation, and external validation cohorts (18). Variables with a *P* < 0.05 in the univariate model were included in the multivariate logistic regression analysis. The measure of the effect of each variable on TE was presented as an odds ratio (OR) to identify independent risk factors. The significance of each variable in the primary cohort was assessed by univariate logistic regression to investigate the independent risk factors for TE in GC patients who received chemotherapy.

All variables with a *P* < 0.05 in the univariate logistic analysis were evaluated by multivariable logistic regression with backward stepwise selection, and the Akaike information criterion was used as a termination rule for the likelihood ratio test (19). According to the results from the final multivariate logistic regression, the nomogram was constructed to visually score individual risk probabilities of TE in GC patients receiving chemotherapy (11, 20).

### The Calibration Curve and Area Under the Receiver Operating Characteristic Curve

We evaluated the calibration of the nomogram by the Hosmer–Lemeshow test and presented it using a calibration curve. The accuracy of the nomogram was presented as a ROC curve, and the area under the receiver operating characteristic curve (AUROC) was used to quantitatively express the ability of the nomogram to predict TE in patients with GC undergoing chemotherapy.

### Clinical Use of the Nomogram

Decision curve analysis (DCA) is a new approach to appraise the potential clinical value of a risk prediction model, which can directly show the potential benefits of the new model once applied in clinical practice (21). Thus, the DCA method was used to compare the clinical consequences of the predictive nomogram in the current research.

## Results

### Demographic and Clinical Characteristics of the Patients

A total of 544 patients were collected in our final study cohort, with 412 and 132 patients assigned to the primary and independent validation cohorts, respectively (Figure 1). The rate of TE was 18.7 and 16.6% in the primary and independent validation sets, respectively (*P* = 0.068). The clinical characteristics of the patients in the primary and independent validation sets are given in Table 1.

**Table 1:** Characteristics of patients in the primary and validation cohorts.

		**Primary set**			**Independent validation set**		
		**TE (+)**	**TE (–)**	***P***	**TE (+)**	**TE (–)**	***P***
Age (mean ± SD, years)		67.1 ± 10.2	67.4 ± 10.5	0.866	70.3 ± 9.3	69.4 ± 9.7	0.670
Gender [*n* (%)]				0.041			0.062
	Male	53 (68.8)	188 (56.1)		15 (68.2)	51 (46.4)	
	Female	24 (31.2)	147 (43.9)		7 (31.8)	59 (53.6)	
TE [*n* (%)]						
	DVT	47 (61.0)	–	–	10 (45.5)	–	–
	PE	10 (13.0)	–	–	7 (31.8)	–	–
	ATE	6 (7.8)	–	–	2 (9.1)	–	–
	PVT	3 (3.9)	–	–	0 (0)	–	–
	DVT + PE	8 (10.4)	–	–	2 (9.1)	–	–
	DVT + ATE	3 (3.9)	–	–	1 (4.5)	–	–
ECOG [*n* (%)]				<0.001			0.035
	0	24 (31.1)	191 (57.0)		4 (18.2)	28 (25.4)	
	1	33 (42.9)	118 (35.2)		12 (54.5)	73 (66.4)	
	2	20 (26.0)	26 (7.8)		6 (27.3)	9 (8.2)	
Histological subtype [*n* (%)]				0.197			0.209
	Well and mod	10 (13.0)	32 (9.6)		22 (100)	96 (87.3)	
	Others	67 (87.0)	292 (87.2)		0 (0)	9 (8.2)	
	Unknown	0 (0)	11 (3.3)		0 (0)	5 (4.5)	
Adj or non-Adj setting [*n* (%)]				0.005			
	Non-Adj	18 (23.4)	136 (40.6)		2 (9.1)	50 (45.5)	0.001
	Adj	59 (76.6)	199 (59.4)		20 (90.9)	60 (54.5)	
Resection of primary site [*n* (%)]				0.542			0.165
	Yes	62 (80.5)	259 (77.3)		19 (86.4)	104 (94.5)	
	No	15 (19.5)	76 (22.7)		3 (13.6)	6 (5.5)	
CVC [*n* (%)]				0.068			0.005
	Yes	29 (37.7)	91 (27.2)		1 (4.5)	38 (34.5)	
	No	48 (62.3)	244 (72.8)		21 (95.5)	72 (65.5)	
Patients with active cancer (AC) [*n* (%)]				<0.001			0.003
	Non-AC	5 (4.5)	107 (31.9)		1 (4.5)	40 (36.4)	
	AC	72 (93.5)	228 (68.1)		21 (95.5)	70 (63.6)	
Khorana score [*n* (%)]				0.850			0.280
	High	28 (36.4)	217 (64.8)		17 (77.3)	72 (65.5)	
	Low	49 (63.6)	118 (35.2)		5 (22.7)	38 (34.5)	
Single or multiple primary [*n* (%)]				0.751			0.956
	Single	68 (88.3)	300 (89.6)		21 (95.5)	105 (95.5)	
	Multiple	9 (11.7)	35 (10.4)		1 (4.5)	5 (4.5)	
D-dimer [*n* (%)]				<0.001			0.002
	<500 μg/L	23 (29.9)	262 (78.2)		8 (36.4)	78 (70.9)	
	≥500 μg/L	54 (70.1)	73 (21.8)		14 (63.6)	32 (29.1)	

*P-value is derived from the univariate association analyses between the TE (+) group and the TE (–) group*.

*TE, thromboembolism; TE (+), TE-positive; TE (–), TE-negative; Adj, adjuvant; PE, pulmonary embolism; DVT, deep vein thrombosis; ATE, arterial thrombosis; PVT, portal vein thrombosis; CVC, central venous catheter; mod, moderately differentiated adenocarcinoma; ECOG, Eastern Cooperative Oncology Group Performance Status; Well, well-differentiated adenocarcinoma*.

### Predictor Selection and Model Development

**Figure 2:**
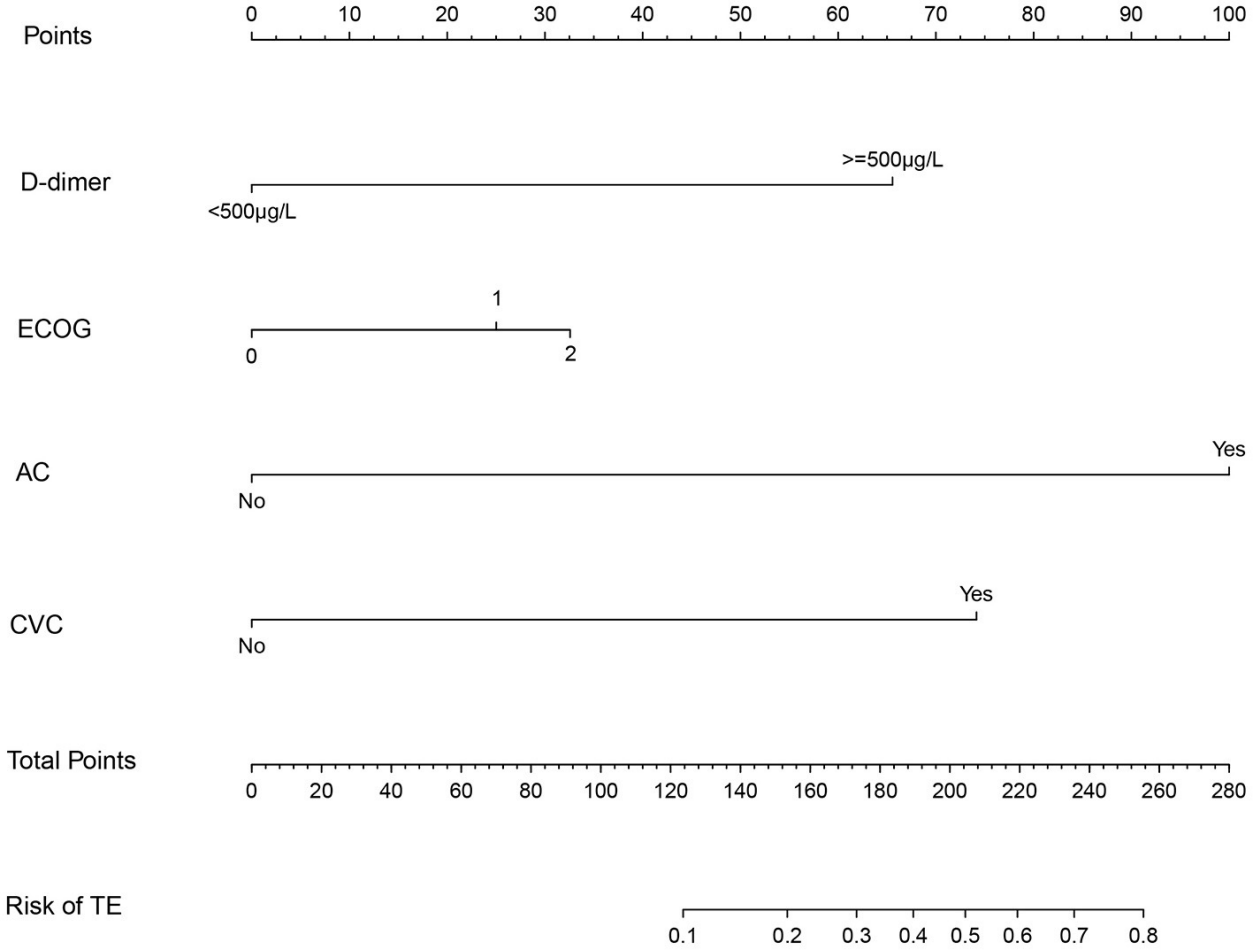
The nomogram model for quantifying individual risk of TE in GC patients who received chemotherapy. For the pretreatment of patients with GC who received chemotherapy, the risk of TE according to the nomogram is the probability in “Risk of TE” corresponding to “Total Points” of all four indicator points summing gastric cancer patients who received chemotherapy.

Patients in the primary study were randomly divided into the training (275 cases) and internal validation sets (137 cases). We evaluated the association between TE and clinicopathological variables. The results of the univariate logistic and multivariate analyses are presented in Table 2. Univariate binary logistic regression analyses showed that the ECOG, the presence of an AC, CVC, and D-dimer levels were significant risk factors for TE in GC patients receiving chemotherapy (*P* < 0.05). According to the multivariate logistic analysis, the results showed that the ECOG [3.233 (0.484–1.863)], AC [47.954 (2.112–5.628)], CVC [9.383 (1.232–3.246)], and D-dimer level [8.136 (1.206–2.987)] were independently associated with TE in GC patients receiving chemotherapy. The model that incorporated the above independent predictors was developed into the nomogram (Figure 2).

**Table 2:** Results of univariate and multivariate analyses for the prediction of incidence of TE.

	**Univariate**			**Multivariate**		
	**β**	***P*-value**	**OR (95% CI)**	**β**	***P*-value**	**OR (95% CI)**
Age (mean ± SD, years)	−0.004	0.785	0.996 (0.967–1.025)			
Gender	−0.514	0.115	0.598 (0.316–1.133)			
ECOG	0.937	0.001	2.551 (0.552–1.321)	1.406	0.001	3.233 (0.484–1.863)
Histological subtype	−0.577	0.169	0.562 (−1.399–0.245)			
Adj or non-Adj setting	0.767	0.028	2.153 (0.083–1.450)	−0.366	0.550	0.693 (0.209–2.299)
Resection of primary site	0.293	0.464	1.341 (0.611–2.942)			
CVC	1.078	0.001	2.940 (0.455–1.701)	2.239	<0.001	9.383 (1.232–3.246)
Patients with active cancer (AC)	1.757	0.001	5.797 (0.699–2.815)	3.870	<0.001	47.954 (2.112–5.628)
Khorana score	−0.603	0.853	0.941 (0.498–1.781)			
Single or multiple primary	−0.587	0.190	0.556 (0.231–1.338)			
D-dimer	1.979	<0.001	7.236 (1.311–2.647)	2.096	<0.001	8.136 (1.206–2.987)

*OR, odds ratio; CI, confidence interval*.

### Performances of Prediction and Calibration

**Figure 3:**
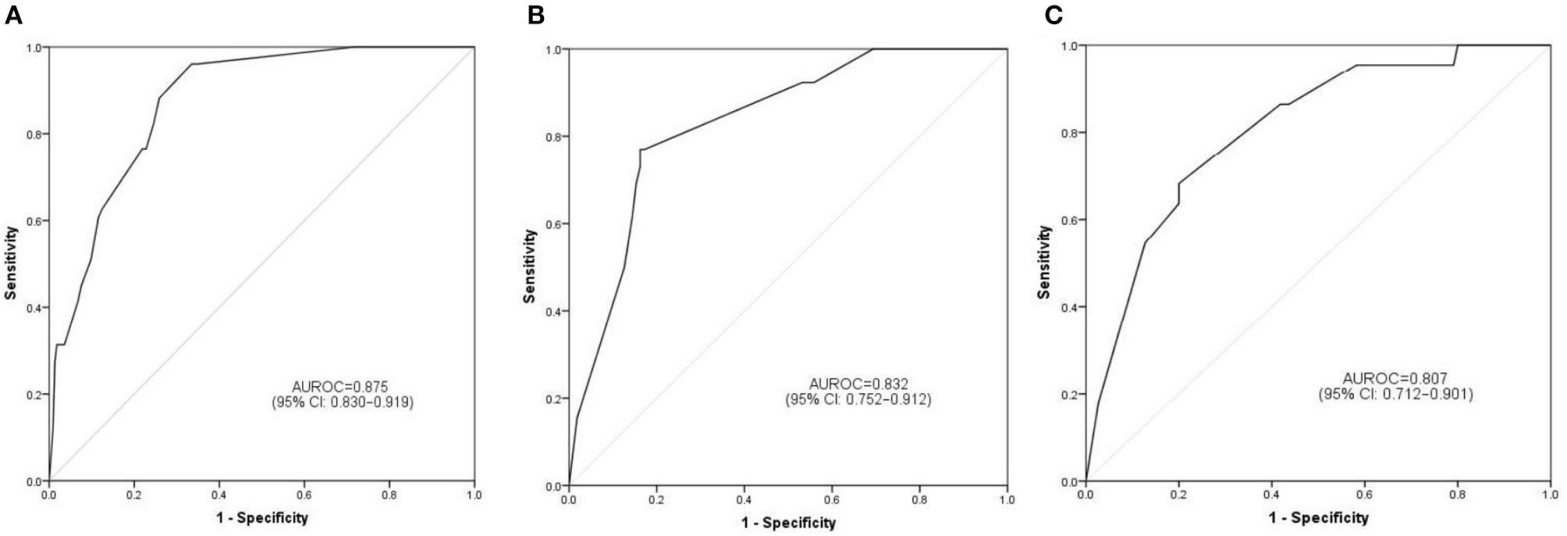
Prediction performance of the model. Receiver operating characteristic (ROC) curve plot in the training set **(A)**; ROC curve plot in the internal validation set **(B)**; ROC curve plot in the independent validation set **(C)**. AUROC, the area under the receiver operating characteristic; CI, confidence interval.

**Figure 4:**
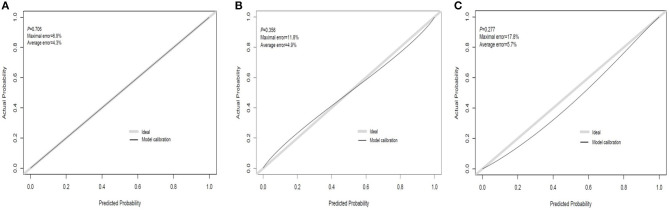
Calibration curve plot in each set. **(A)** The training set; **(B)** the internal validation set; **(C)** the independent validation set.

The discrimination ability and prediction performance of the nomogram were represented by the ROC curve (Figure 3). The nomogram demonstrated good valuable prediction performance with an AUROC of 0.875 (0.832 in the internal validation and 0.807 in the independent validation, respectively). The calibration curves of the nomogram showed a good agreement between prediction and observation (Figure 4). We obtained a good calibration curve in the nomogram and the Hosmer–Lemeshow test was not significant in each set (*P* > 0.05), which indicated a high reliability of the nomogram's prediction ability.

### Presentation of the Nomogram and Clinical Risk Management

**Figure 5:**
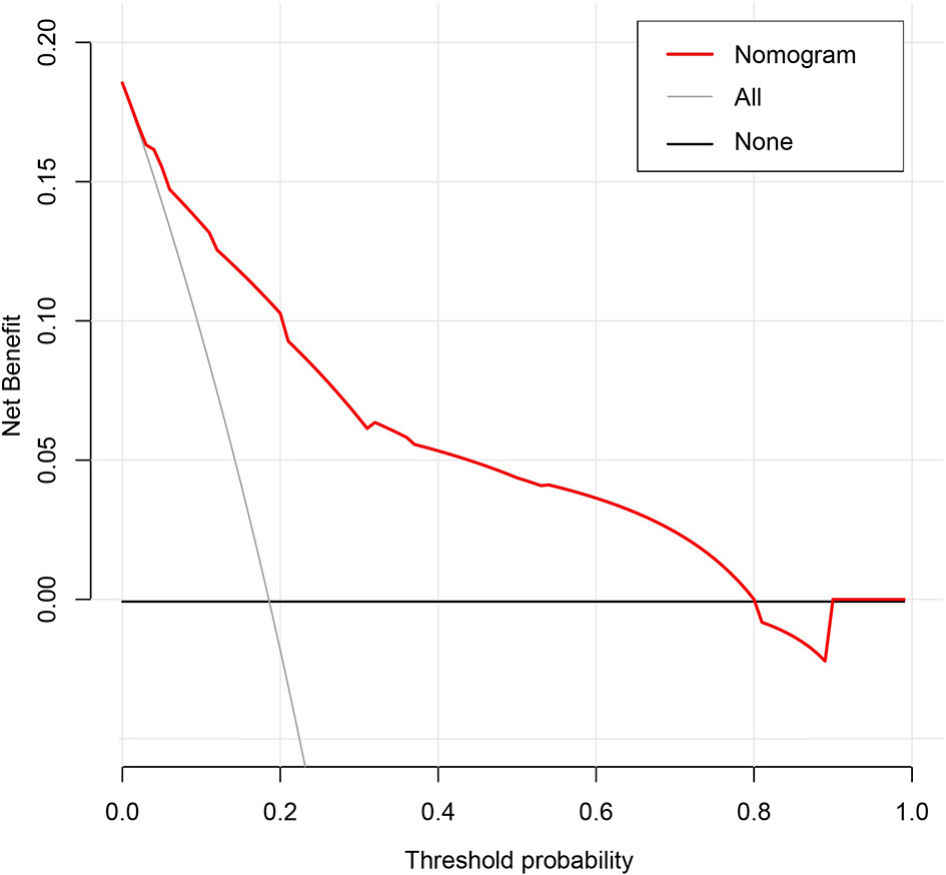
Decision curve analysis for the classification of different risk populations.

The results of the DCA for the nomogram are presented in Figure 5. The decision curve of the net benefit showed a superior risk threshold probability to the baseline, ranging from 6.1 to 80%. If the threshold probability was 10%, the net benefit was 0.135 superior to the treatment-all of 0.117 and treatment-none, and if the risk threshold probability was 5% (<6%) and 85% (>80%), the net benefit of 0.152 and −0.016 is not superior to the reference strategies of treatment-all of 0.185 and treatment-none, respectively.

## Discussion

Early prediction of TE is important to improve the quality of life of patients with GC receiving chemotherapy. In this study, we developed and validated a simple prediction model based on four clinical indicators to quantify the risk of TE in patients with GC after chemotherapy, which can be used by clinicians for the individualized risk management of TE in patients with GC after chemotherapy. To the best of our knowledge, this study was the first to use a nomogram in TE in GC patients who received chemotherapy based on large-scale multicentric datasets including 544 patients. The easy-to-use nomogram contains four clinical risk factors (ECOG, AC, CVC, and D-dimer) to predict the risk of thrombosis in GC patients receiving chemotherapy.

Various risk factors for cancer-related TE have been previously reported, such as age, histological subtype, stage, and chemotherapy (7, 22). Certain cancers have also been identified as high risk factors for TE, including lung cancer, pancreatic cancer, and gastric cancer (23–25). To best of our knowledge, few studies have developed or validated risk prediction models for TE in cancer patients receiving chemotherapy. The Khorana score is a predictive risk assessment model for TE in cancer patients (10). Sanfilippo et al. (26) developed and validated a risk score to assess the risk of patients with multiple myeloma starting chemotherapy, with an area under the curve value of 0.66.

Active cancer is associated with an increased TE risk with an overall four- to seven-fold increased risk (27). Compared with cancer patients without distant metastases, patients with distant metastases have a higher risk of TE (5, 28). Cancer-associated TE is associated with biological invasiveness of tumors, as the pathways of coagulation and fibrinolysis intersect with those of tumor growth and metastasis (29, 30). Aggressive tumors grow faster and are more likely to metastasize and spread, leading to a higher risk of TE (31). In this study, active cancer was associated with a higher incidence of TE, suggesting that both distant metastasis and early recurrence reflect tumor invasiveness.

Central venous catheterization (CVC), including PICC, has become a common way of infusion. Although CVC has the advantages of safety and convenience, the complications of CVC represented by CVC-related thrombosis have also been reported (32). The incidence of PICC-related thrombosis is closely related to the type of central venous catheter (33). In addition, the incidence of TE is related to the thickness of the catheter; the thicker the central venous catheter, the higher the incidence of VTE, and the more serious the damage to the vascular epidermis (32, 33). Ten et al. (34) found that the risk of VTE was different when CVC was carried out at different sites. The incidence of TE in patients with left arm puncture was significantly higher than that in patients with right arm puncture and could be related to the distance between left arm puncture point and superior vena cava. In addition, the depth of the CVC catheter placement was associated with the incidence of VTE (33).

Plasma D-dimer levels have been identified as a predictive biomarker of TE, but its specificity is not high. Increased levels of plasma D-dimer have also been associated with pregnancy, surgery, inflammation, infection, and various types of cancer (35, 36). D-dimer levels are elevated in patients with ovarian cancer after surgery and have been recommended as a predictor of thrombosis in patients (37, 38). According to previous studies, the threshold of D-dimer for predicting thrombosis is still controversial. According to the receiver operating characteristic curve, we found that the optimal critical level of plasma D-dimer to distinguish the thrombus group from the non-thrombus group was 500 ng/ml. This is similar to that after lung cancer surgery (39). The critical D-dimer plasma level in GC patients undergoing chemotherapy was 500 μg/L, which requires further consideration and research. In addition, false-positive increases in D-dimer levels are common in cancer patients. Therefore, the specificity and positive predictive value of the assay are likely to be reduced in cancer patients.

The ECOG is a method to evaluate the functional performance status of patients. Performance status is an important indicator of activities of daily living of cancer patients (40). Performance status has been repeatedly demonstrated in most studies to predict the clinical outcomes of cancer patients, including the quality of life, chemotherapy toxicity, response to chemotherapy, and overall survival (41–43). Most studies have shown that the ECOG is closely related to the prognosis of cancer patients receiving chemotherapy (44–46). Compared with patients with an ECOG of 0–1, patients with a higher score (ECOG ≥ 2) generally had poorer tolerance to chemotherapy (47–49). However, in daily clinical practice, patients with an ECOG ≥ 3 do not often receive chemotherapy (50). In our study, the vast majority of GC patients who received chemotherapy scored <2. Interestingly, a recent clinical study showed that the ECOG was independently associated with TE in Japanese gastric and colorectal cancer (GCC) patients who received chemotherapy (51).

Our nomogram only contained four variables, which represented a simple and visual tool for the risk probability of venous thrombosis in GC patients undergoing chemotherapy. This study shows that this simple risk assessment model based on four clinical indicators can reliably predict the risk of TE in patients with GC at the beginning of chemotherapy. TE is a frequent occurrence in GC patients undergoing chemotherapy, and it can be prevented by effective anticoagulant therapy. This predictive model can be used by clinicians to assess the risk of TE in patients with GC undergoing chemotherapy in clinical practice and can also be used to design future clinical trials involving cancer patients who will benefit from thromboprophylaxis.

The most important issue in this model is the individual needs for anticoagulation in GC patients receiving chemotherapy. Although the nomogram has a better risk prediction performance, calibration, and resolution, it still cannot capture the clinical consequences of a certain level of discrimination or misalignment (52–54). Therefore, in order to confirm its clinical application, decision curve analysis was used to evaluate whether the decision-making based on the nomogram was helpful. The decision curve shows that the model has a positive net income for threshold probabilities between 6.1 and 80%. For example, if the personal threshold probability is 10%, the net benefit is 0.135 superior to the treatment-all of 0.117 and treatment-none when using the nomogram to decide whether to conduct anticoagulation.

This study has limitations. First of all, this study used a retrospective analysis, and the underlying bias could not be avoided. Therefore, the reliability and stability of the nomogram still need to be further verified. In addition, the sample size of this study is not very large, and the samples are all from the same country and race. Whether, the model is suitable for patients of other races and countries is unknown. Finally, further prospective multicenter clinical studies are needed to prove its clinical efficacy.

### Conclusion

This study systematically developed and validated a novel nomogram model for predicting TE in patients with GC receiving chemotherapy. With this easy-to-use scoring system, physicians could perform pretreatment of TE management, facilitating timely individualized clinical decision-making for different risks in patients with GC receiving chemotherapy.

## Data Availability Statement

The raw data supporting the conclusions of this article are available from the corresponding author prior to approval from the Ethics Committee.

## Ethics Statement

This retrospective study was approved by the Ethics Committee of the First Affiliated Hospital of Nanchang University and the Ethics Committee of the Beilun branch Ethics Committee of the First Affiliated Hospital of Zhejiang University, respectively. The requirement for patient-informed consent was waived because of the retrospective nature of this study, and the patients' data confidentiality was protected.

## Author Contributions

H-LY and J-JL contributed to the idea and design. H-LY, XZ, YL, and J-JL contributed to the data acquisition and analysis. H-LY, XZ, and J-JL contributed to the manuscript writing and revision. All authors contributed to data acquisition and analysis and to manuscript writing and revision and agreed to all aspects of the work.

## Conflict of Interest

The authors declare that the research was conducted in the absence of any commercial or financial relationships that could be construed as a potential conflict of interest.
